# Immunohistochemical PSMA expression patterns of primary prostate cancer tissue are associated with the detection rate of biochemical recurrence with ^68^Ga-PSMA-11-PET

**DOI:** 10.7150/thno.44584

**Published:** 2020-05-15

**Authors:** Daniela A. Ferraro, Jan H. Rüschoff, Urs J. Muehlematter, Benedikt Kranzbühler, Julian Müller, Michael Messerli, Lars Husmann, Thomas Hermanns, Daniel Eberli, Niels J. Rupp, Irene A. Burger

**Affiliations:** 1 Department of Nuclear Medicine, University Hospital Zürich, University of Zürich, Zurich, Switzerland; 2 Department of Pathology and Molecular Pathology, University Hospital Zurich, University of Zurich, Zurich, Switzerland.; 3 Department of Interventional and Diagnostic Radiology, University Hospital Zurich, University of Zurich, Zurich, Switzerland; 4 Department of Urology, University Hospital Zürich, University of Zurich, Zurich, Switzerland; 5 Department of Nuclear Medicine, Kantonsspital Baden, Baden, Switzerland

**Keywords:** immunohistochemistry, PSMA-negative prostate cancer, PSMA PET, PSMA staining, restaging

## Abstract

Prostate-specific membrane antigen (PSMA) targeted PET has a high detection rate for biochemical recurrence (BCR) of prostate cancer (PCa). Nevertheless, even at high prostate-specific antigen (PSA) levels (> 3 ng/ml), a relevant number of PSMA-PET scans are negative, mainly due to PSMA-negative PCa. Our objective was to investigate whether PSMA-expression patterns of the primary tumour on immunohistochemistry (IHC) are associated with PSMA-PET detection rate of recurrent PCa.

**Methods:** Retrospective institutional review board approved single-centre analysis of patients who had undergone ^68^Ga-PSMA-11-PET for BCR after radical prostatectomy (RPE) between 04/2016 and 07/2019, with tumour specimens available for PSMA-IHC. Clinical information (age, PSA-level, ongoing androgen deprivation therapy (ADT), Gleason score) and PSMA-IHC of the primary tumour were collected and their relationship to results from PSMA-PET (positive/negative) was investigated using a multiple logistic regression analysis.

**Results:** 120 PSMA-PET scans in 74 patients were available for this analysis. Overall detection rate was 62% (74/120 scans), with a mean PSA value at scan time of 0.99 ng/ml (IQR 0.32-4.27). Of the clinical factors, only PSA-level and ADT were associated with PSMA-PET positivity. The percentage of PSMA-negative tumour area on IHC (PSMA_%neg_) had a significant association to PSMA-PET negativity (OR = 2.88, *p* < 0.001), while membranous PSMA-expression showed no association (*p* = 0.73). The positive predictive value of PSMA_%neg_ ≥ 50% for a negative PSMA-PET was 85% (13/11) and for a PSMA_%neg_ of 80% or more, 100% (9/9).

**Conclusions:** PSMA-negative tumour area on IHC exhibited the strongest association with negative PSMA-PET scans, beside PSA-level and ADT. Even at very high PSA levels, PSMA-PET scans were negative in most of the patients with PSMA_%neg_ ≥ 50%.

## Introduction

Prostate-specific membrane antigen (PSMA) is a type 2 integral membrane protein expressed in the cytoplasm of normal prostate tissue and particularly overexpressed on the cell membrane in prostate cancer (PCa) 1-4. There is increasing evidence that PSMA-expression of the primary tumour is associated with a higher Gleason Score (GS) and a worse prognosis 5-10. Recently, higher membranous PSMA-expression was not only associated with hormone resistant PCa, but also with an increase in defective DNA repair mutations, a further explanation why PSMA-expression seems to have a strong association to survival 9. However, PSMA-expression is very heterogeneous with a marked inter- and intrapatient heterogeneity 1. Furthermore, increasing evidence shows that even within the same primary tumour PSMA-expression can be highly variable 11. Therefore, the association between PSMA-expression of primary tumour and metastases is still not well understood. However, it is reasonable to hypothesize that metastasis of patients with a PSMA-negative primary tumour are more likely to also be PSMA-negative. Interestingly, in a recently published paper Paschalis et al. found that absence of PSMA-expression in the primary lesion on immunohistochemistry (IHC) at diagnosis was associated with a lack of PSMA expression on IHC in the metastasis in patients with castration-resistant disease 9.

PSMA expression in PCa has gained great importance in the past decade since PSMA binding tracers are increasingly used for positron emission tomography (PET) 12. In patients with biochemical recurrence (BCR), PSMA-PET holds a high detection rate with an impact on disease management in around 50% of patients 13-15. Detection and localization of lesions depend on prostate-specific antigen (PSA) level, with patient-based detection rates of around 50% with PSA levels ≤ 0.5 ng/ml and around 90% with PSA levels > 3 ng/ml 16, 17.

According to the literature, around 5-10% of primary PCa are PSMA-negative on IHC 8 and around 10% are negative on PSMA-PET despite high PSA levels 18, 19. Even though PSMA-negative primary PCa is associated with a better prognosis 5, 8, 20, PSMA-negative recurrence represents a challenge due to false negative PSMA-PET scans. In some cases, after a first negative PSMA-PET, clinician and patient opt to postpone treatment initiation in attempt to localize disease in a subsequent examination 15, 21, 22. Therefore, discrimination between negative PSMA-PET due to its limited spatial resolution of small tumour burden or due to PSMA-negative PCa could improve patient management.

The aim of our study is to investigate whether the immunohistochemical PSMA expression patterns on IHC of the primary tumour is associated with the ^68^Ga-PSMA-11-PET positivity for BCR.

## Patients and Methods

### Study population

This retrospective single-centre cohort study included all consecutive patients referred to ^68^Ga-PSMA-11-PET for BCR of PCa or PSA persistence after surgery between April 2016 and July 2019 who had previously undergone radical prostatectomy (RPE) at our institution. For patients who had had more than one ^68^Ga-PSMA-11-PET for restaging, up to 3 scans were included per patient, including subsequent scans after a previously negative ^68^Ga-PSMA-11-PET and further scans because of a new rise in PSA value after ^68^Ga-PSMA-11-PET-guided treatment. ^68^Ga-PSMA-11-PET for staging PCa were excluded, as well as patients with prostatectomy specimen not available for histopathological evaluation. For patients with nodal metastasis found by pelvic lymph node dissection (pLND), the largest nodal metastasis was included in histopathology analysis. The local ethics committee approved the study protocol and all patients had given a general written informed consent for retrospective use of their data (BASEC Nr. 2016-01776).

### Study design

We collected relevant clinical data from patient's charts such as PSA level at scan time, tumour (modified) Gleason Score (GS) / respective ISUP/WHO prognostic grade group 23 and information regarding ongoing androgen deprivation therapy (ADT). Prostatectomy specimens were analysed for PSMA expression in the primary tumour on IHC. We classified ^68^Ga-PSMA-11-PET for BCR as positive or negative according to the clinical reports. For the positive scans, maximum standard uptake value (SUV_max_) was recorded for local recurrence and for the lesion with the highest SUV_max_ in case of metastasis, which were also characterized by localization (local lymph nodes (LN), distant LN, bone or visceral).

### Imaging

Patients had undergone clinical routine ^68^Ga-PSMA-11-PET/computed tomography (CT) on a Discovery VCT 690 PET/CT (GE Healthcare, Waukesha, WI, USA) or on a Discovery MI PET/CT (GE Healthcare, Waukesha, WI, USA) or ^68^Ga-PSMA-11-PET/magnetic resonance (MR) (SIGNA PET/MR, GE Healthcare, Waukesha, WI, USA) after a single injection of ^68^Ga-PSMA-11 (mean dose ± standard deviation (SD) 130 ± 18 MBq, range 81-171 MBq). The institutional protocol is in agreement with the EANM and SNMMI procedure guidelines 24. Details are given in the supplements.

#### Imaging analysis

The acquired PET/CT and PET/MR images were analysed in a dedicated review workstation (Advantage Workstation, Version 4.6 or 4.7, GE Healthcare), which enables the review of the PET and the CT or MR images side by side and in fused mode. All scans were analysed and reported on clinical routine by dual board-certified radiologists and nuclear medicine physicians with 5-10 years of experience, incorporating both the MR or CT and PET information and being aware of ^68^Ga-PSMA-11-PET pitfalls such as neural ganglia, Paget's disease, sarcoidosis and others 25, 26. In order to correctly classify ^68^Ga-PSMA-11-PET scans as positive or negative the validated Prostate Cancer Molecular Imaging Standardized Evaluation (PROMISE) guidelines were used 27. Patients with equivocal findings on reports were followed-up to determine whether lesions were true or false-positive. SUV_max_ was recorded for all local recurrence lesions as well as for the metastatic lesion with the highest SUV_max_ in each positive ^68^Ga-PSMA-11-PET.

### Radical prostatectomy and lymphadenectomy

All RPEs were performed in form of a robot-assisted transperitoneal laparoscopic radical prostatectomy including the seminal vesicles, with bilateral pLND by experienced urologists at our institution as described earlier 28. All operations were performed using the four-arm Da Vinci SI system (Intuitive Surgical, Inc., USA). pLND included the external iliac, obturator and internal iliac LN with an upper resection boundary defined by the crossing of the ureter over the common iliac artery and was performed in all patients selected for RPE.

### PSMA and additional staining on immunohistochemistry

One slide from the RPE specimen was chosen for further investigation, harbouring the largest area of representative tumour and therefore defining the dominant tumour. For the regional lymph node metastases (pN1) the single one or, if more than one was available, the largest one was chosen for further investigation**.** PSMA-IHC staining for the dominant tumour on the prostatectomy specimen and for the largest metastatic node from pLND was performed as described previously 29. The predominant PSMA-expression patterns were visually quantified using a four-tiered system (0 = negative, 1+ = weak, 2+ = moderate, 3+ = strong) for each membranous and cytoplasmic PSMA expression (PSMA_memb_ and PSMA_cytosol_) by two board certified, experienced genito-urinary pathologists (J.H.R, N.J.R.). Examples of expression patterns are shown in Figure S1 (supplementary material). Furthermore, tumour areas without PSMA expression were quantified in steps of 5%, 10% and further 10% increments in relation to the total tumour area, as percentage PSMA-negative tumour area (PSMA_%neg_) as a consent of both pathologists. Heterogeneity was defined by differences in the staining pattern of at least 5% of the representative tumour slide, in both primary tumour and lymph node metastasis (Figure S2, supplementary material).

In selected cases, depending on the results, additional stainings were performed. For Chromogranin A staining, the LK2H10 clone (1:500, Cell Marque Lifescreen Ltd.) was used like previously described PSMA staining. For Synaptophysin staining the 27G12 clone (1:50, Novocastra Ltd.) and for Androgenreceptor the F39.4.1 clone (1:250, BioGenex) were used on a Leica Bond device according to standard protocols. Synaptophysin expression was visually evaluated in 1%, 5%, 10% and further 10% steps of expression. Predominant Androgenreceptor expression of the tumour was semi-quantitatively evaluated using a four-tiered system (0 = negative, 1+ = weak, 2+ = moderate, 3+ = strong).

### Statistical analysis

Descriptive statistics were used to display patient data as median and interquartile range (IQR) with 25^th^ and 75^th^ percentiles (Q1-Q3), as well as percentages, and were performed using Excel (Excel, version 2016, Microsoft, USA). Data was analysed for normal distribution using normal probability plots. Correlations were done using bivariate Pearson's correlation in SPSS Version 25 (IBM, Armonk, New York, USA) and results are presented with the coefficient (r), *p* value and confidence interval (CI). Receiver operating curves (ROC) were analysed using DeLong method in Medcalc Statistical Software version 18.2.1 (MedCalc Software bvba, Ostend, Belgium).

Associations between ^68^Ga-PSMA-11-PET positivity and several clinical and primary tumour variables were assessed and investigated with multiple logistic regression analysis. The variables entered in the multiple logistic regression analysis were selected by a univariate analysis with a *p* value cut-off point of 0.1. All logistic regression analyses were performed using R (details given in the supplements, R version 3.6.1; R Foundation for Statistical computing, Vienna, Austria) and SPSS Version 25 (IBM, Armonk, New York, USA). A *p* value of < 0.05 was considered statistically significant. All tests were two-tailed.

## Results

**Figure 1:**
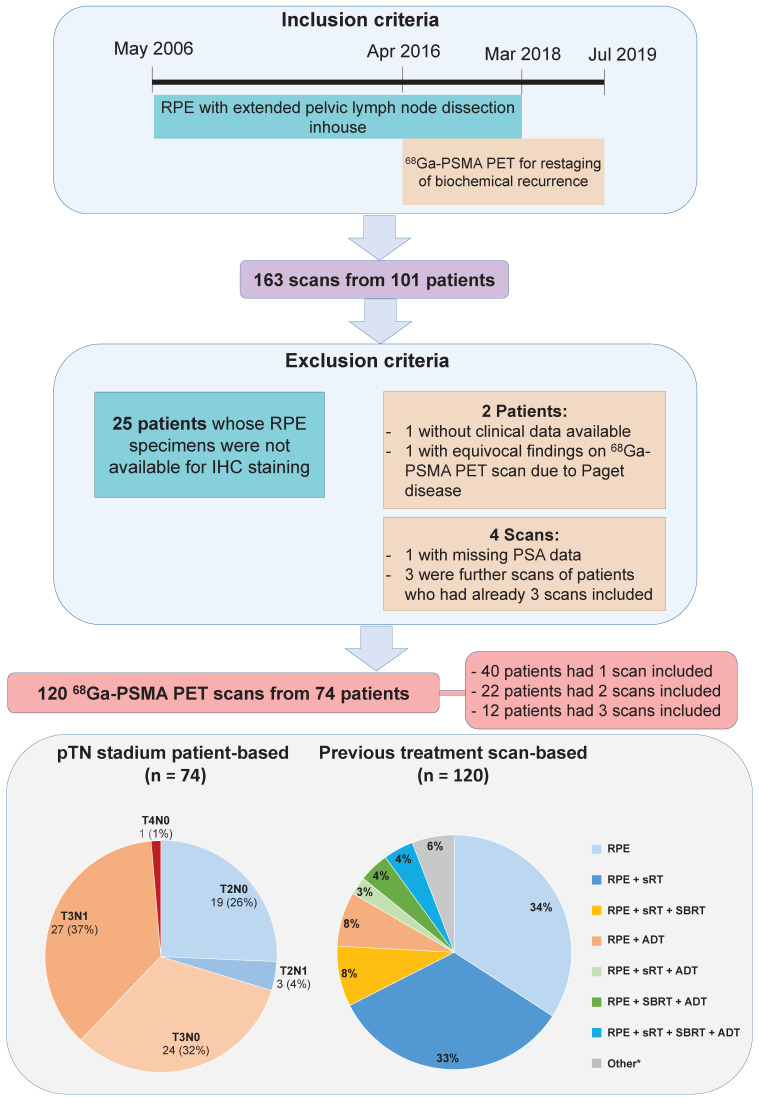
Patient inclusion flowchart and patient characteristics. *including salvage lymph node dissection and other treatment combinations. *ADT = androgen-deprivation therapy; IHC = immunohistochemistry; RPE = radical prostatectomy; SBRT = stereotactic body radiation therapy; sRT = salvage radiotherapy*

One hundred and sixty-three ^68^Ga-PSMA-11-PET scans from 101 patients were available. Patients or scans were excluded because of unavailability of clinical data or RPE specimens as well as the fourth scan of patients who already had three scans included. A total of 120 ^68^Ga-PSMA-11-PET scans from 74 patients were available for this retrospective analysis. Figure 1 illustrates patient selection and characteristics. Interval between surgery and ^68^Ga-PSMA-11-PET ranged from two months to 13 years (median 4 years). Seven scans, in seven patients, were performed for PSA persistence after RPE with intervals from 2 to 4.2 months. In 30 patients there was pN1 disease on pLND. All patients were classified as having high-risk disease according to D'Amico risk score, based on initial PSA level and pT stage and GS after surgery. Characteristics of the patients at staging and at BCR are shown in Table 1.

**Figure 2:**
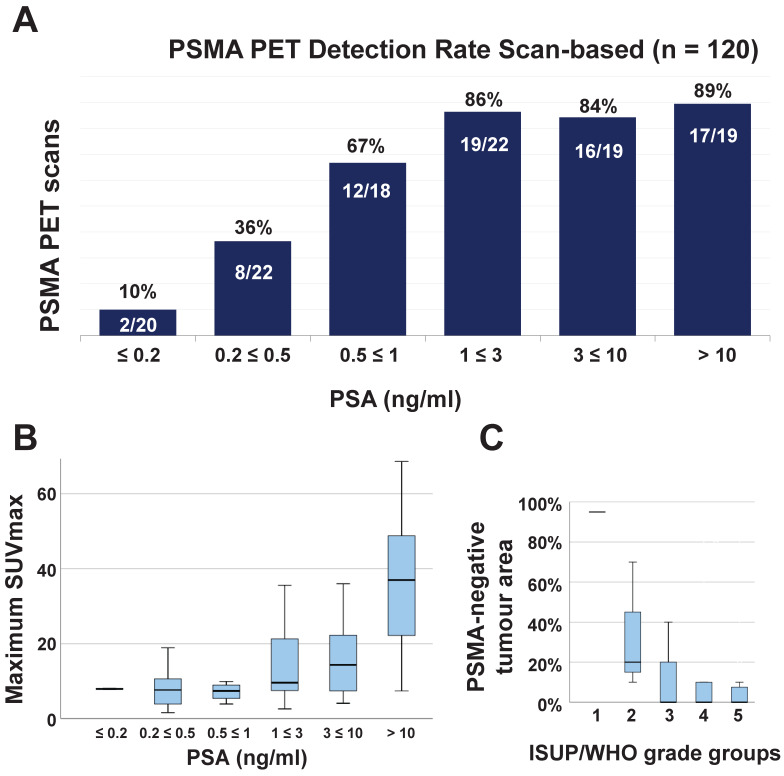
** (A)** Bar chart of 68Ga-PSMA-11-PET detection rate in relation to the PSA level at scan time. **(B)** Boxplot of the correlation between patient PSA level at scan time and maximum SUV_max_ of ^68^Ga-PSMA-11-PET for the 74 positive scans (r = 0.518, *p* < 0.001, CI: 0.260, 0.594). **(C)** Boxplot of the inverse correlation between PSMA-negative tumour area on immunohistochemistry (PSMA_%neg_) and maximum ISUP/WHO grade groups of the primary tumour (r = -0.243, *p* < 0.007, CI: -0.066, -0.420) for all 74 patients included in the study. *SUV_max_ = maximum standardized uptake value*

Median PSA value at scan time was 0.99 ng/ml (IQR 0.32-4.27, mean 8 ng/ml). Seventy-four of the 120 (62%) ^68^Ga-PSMA-11-PET scans were positive. In 10 scans (13%, 10/74), local recurrence was detected (median SUV_max_ 10.2, IQR 7.5-14.3), in 4 of them (5%, 4/74) it was the only suspicious lesion for PCa. In 70 scans, metastatic lesions were detected (median SUV_max_ 9.7, IQR 7.4-24.1, considering the lesion with the highest SUV_max_). The highest SUV_max_ was found in the prostate bed in 9 scans (12%, 9/74), in nodes in 33 scans (45%, 15 pelvic, 18 distant), in bone in 27 scans (36%) and in visceral organs in 5 (7%, 4 in the lungs and 1 peritoneal). Figure 2 (A and B) shows the ^68^Ga-PSMA-11-PET detection rate according to PSA level and the correlation between PSA and SUV_max_.

From the 30 patients with pN1 disease (from the pLND), a total of 44 scans was available. Twenty-eight scans (64%) were positive and 16 (36%) were negative. All positive scans showed PSMA positive metastases with additional local recurrence only in one.

### PSMA-expression on IHC and additional staining

#### Primary tumour

Forty primary tumours (54%, 40/74) fully expressed PSMA (PSMA_%neg_ = 0) and 34 showed some PSMA-negative area: in 25 PSMA_%neg_ was < 50% (34%, 25/74) and in 9 ≥ 50% (12%). The primary tumour showed a homogeneous PSMA expression in 25 specimens (34%) and heterogeneous in 49 (66%). PSMA-expressing tumour area correlated with GS (r = 0.243, *p* = 0.007, CI: 0.066, 0.420, Figure 2C) and intensity of PSMA expression for both PSMA_cytosol_ (r = 0.328, *p* < 0.001, CI: 0.156, 0.501) and PSMA_memb_ (r = 0.583, *p* < 0.001, CI: 0.434, 0.731). PSMA_cytosol_ and PSMA_memb_ had a positive correlation with each other (r = 0.374, *p* < 0.001, CI: 0.205, 0.543).

**Figure 3:**
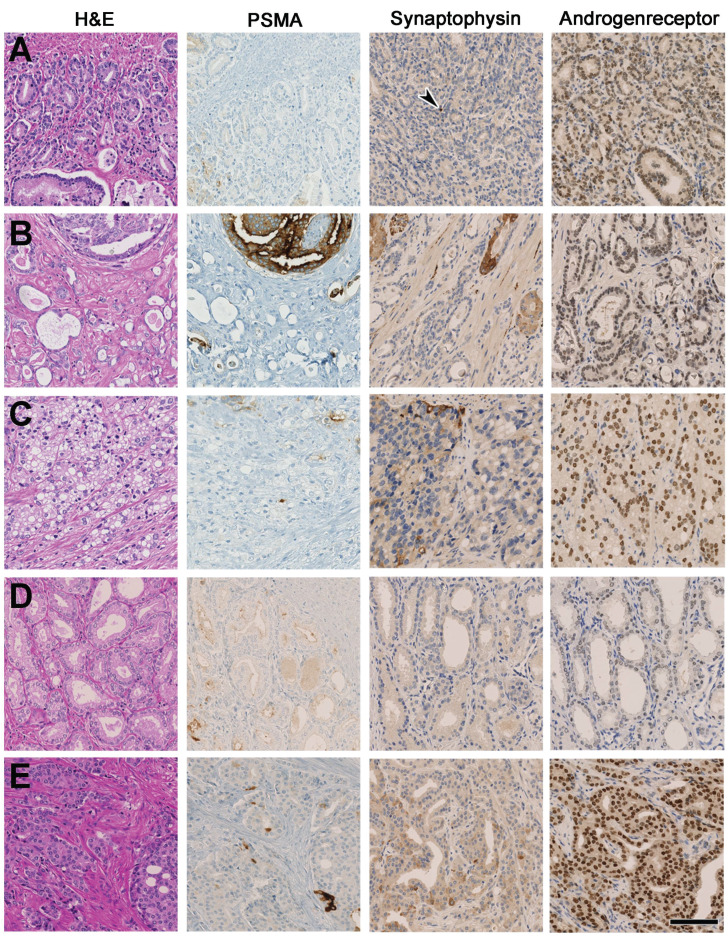
Overview of the primary tumour of the 5 patients with PSMA_%neg_ > 50%. **(A)** shows GS 4+4 = 8, poorly defined, fused glands, wide negativity for PSMA, only single cell expression of Synaptophysin (arrowhead) and moderate Androgenreceptor (AR) expression (Pat. 14). **(B)** shows GS 4+4 = 8, focal cribriform morphology in H&E and predominantly fused glands with secretions, focal expression of PSMA and single cell expression of Synaptophysin and moderate AR experssion (Pat. 59). **(C)** shows GS 4+5 = 9, poorly defined to solid glands with cytoplsamic vacuoles, vast negativity in the PSMA staining, single cells reactive for synaptophysin and moderate AR expression (Pat. 19). **(D)** shows GS 3+3 = 6, isolated glands can be appreciated being widely negative for PSMA, with no Synaptophysin expression and weak AR reactivity (Pat. 31). **(E)** shows GS 4+5 = 9, poorly defined glands and focal cribriform growth is visiable with vast negativity for PSMA, single cell Synaptophysin expression and strong AR reactivity (Pat. 62). Scale bar 100 mu.

The primary tumours from patients with PSA > 1 ng/ml and PSMA_%neg_ > 50% (8 scans from 5 patients) were re-evaluated on pathology. The morphology showed mixed conventional patterns (Table 2; Figure 3), without morphological evidence of a large- or small cell neuroendocrine carcinoma. Also staining for the neuroendocrine marker Synaptophysin revealed only single cell reaction at a maximum of < 5% of the tumour cells. Identical Synaptophysin expression patterns were obtained in a control group of 6 carcinomas with strong and homogenous PSMA-expression correlating with positive ^68^Ga-PSMA-11-PET scans.

#### Lymph nodes

**Figure 4:**
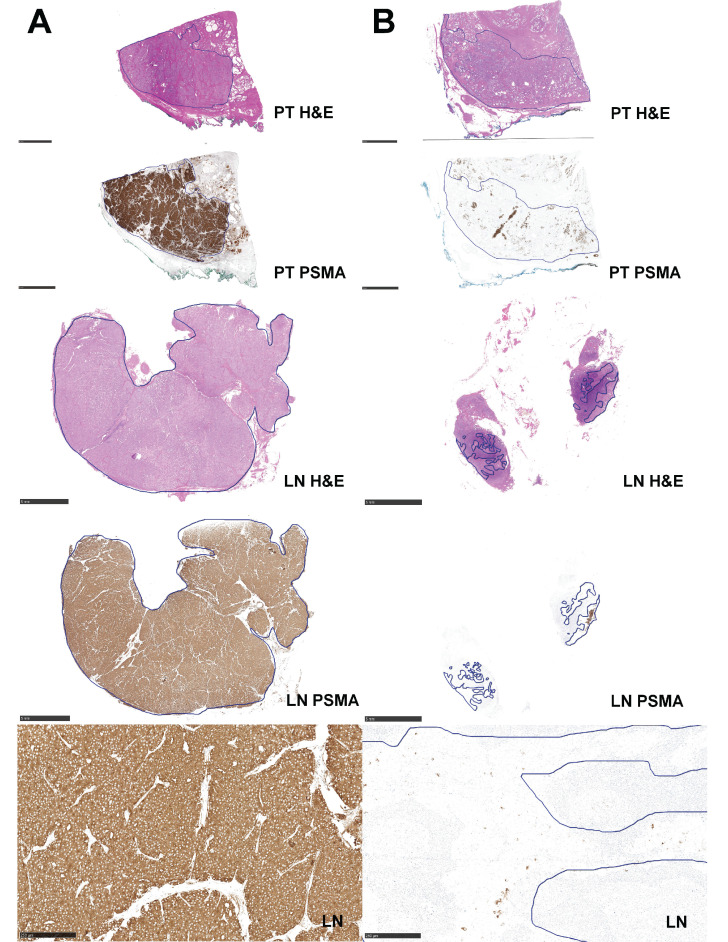
Hematoxylin and eosin staining (H&E) and immunohistochemical (IHC) PSMA staining of primary tumour (PT) and metastatic lymph node (LN) from two patients who had LN showing a homogeneous pattern of PSMA-expression and PSMA_%neg_ in concordance to the PT. **(A)** Shows a PT with GS 4+4 = 8 and 0% PSMA_%neg_ and LN metastasis with GS 4+5 = 9 and 0% PSMA_%neg_ (Pat.45). **(B)** Shows a primary tumour with GS 4+4 = 8 and 80% PSMA_%neg_ and LN metastasis with GS 4+5 = 9 and 90% PSMA_%neg_ (Pat.45). Bars represent 5mm with tumour outlined in blue, with exception of the bottom row which shows a magnification of PSMA-IHC images (bar 250 µm).

PSMA-expression was more homogeneous in LN compared to primary tumours, with 22 homogenous (73%, 22/30) compared to only 8 heterogeneous nodes (7%). 25 nodes (83%, 25/30) showed no PSMA_%neg_ (Figure 4A). Just two nodes had more than 50% PSMA_%neg_ (Figure 4B). The two lymph node metastases in Figure 3 were stained for neuroendocrine markers such as Synaptophysin and Chromogranin A and revealed a maximum of < 1% reactive cells in these stainings. A positive correlation was found between primary tumour and nodes for both PSMA_%neg_ (r = 0.841, *p* < 0.001, CI: 0.731, 1) and PSMA_memb_ (r = 0.446, *p* = 0.002, CI: 0.146, 0.616). Table 3 gives a patient-based comparison between PSMA-IHC of the primary tumour and the LN, as well as an overview of the positive and negative ^68^Ga-PSMA-11-PET scans according to PSA level for these 30 patients.

### Logistic regression analysis

All predictors except PSA showed an approximately normal distribution. Since PSA showed a skewed distribution and suspected nonlinear effect has been reported 30, a log transformation was applied to this predictor. In the univariate analysis to predict negative scans (results given in the supplements), age, injected tracer dose and ISUP grade group did not reach the cut-off point of *p* = 0.1 and were, therefore, not selected for the multiple logistic regression. In the multiple logistic regression analysis, only PSA, ongoing ADT and PSMA_%neg_ had an association with ^68^Ga-PSMA-11-PET detection rate. Both higher PSA values and ongoing ADT were associated with scan positivity yielding areas under the ROC curve (AUC) of 0.836 (95% CI: 0.757-0.914, *p* < 0.001 / OR 0.28, *p* < 0.001) and 0.736 (95% CI: 0.648-0.823, *p* < 0.001 / OR 0.01, *p* = 0.047), respectively. PSMA_%neg_ of the primary tumour was associated with scan negativity with an AUC of 0.608 (95% CI: 0.501-0.714, *p* = 0.047 / OR 2.88, *p* < 0.001). PSMA_cytosol_ and PSMA_memb_ had no association with ^68^Ga-PSMA-11-PET detection rate. Values for all variables are given in Table 4.

### Correlation of PSMA_%neg_ with ^68^Ga-PSMA-11-PET positivity

**Figure 5:**
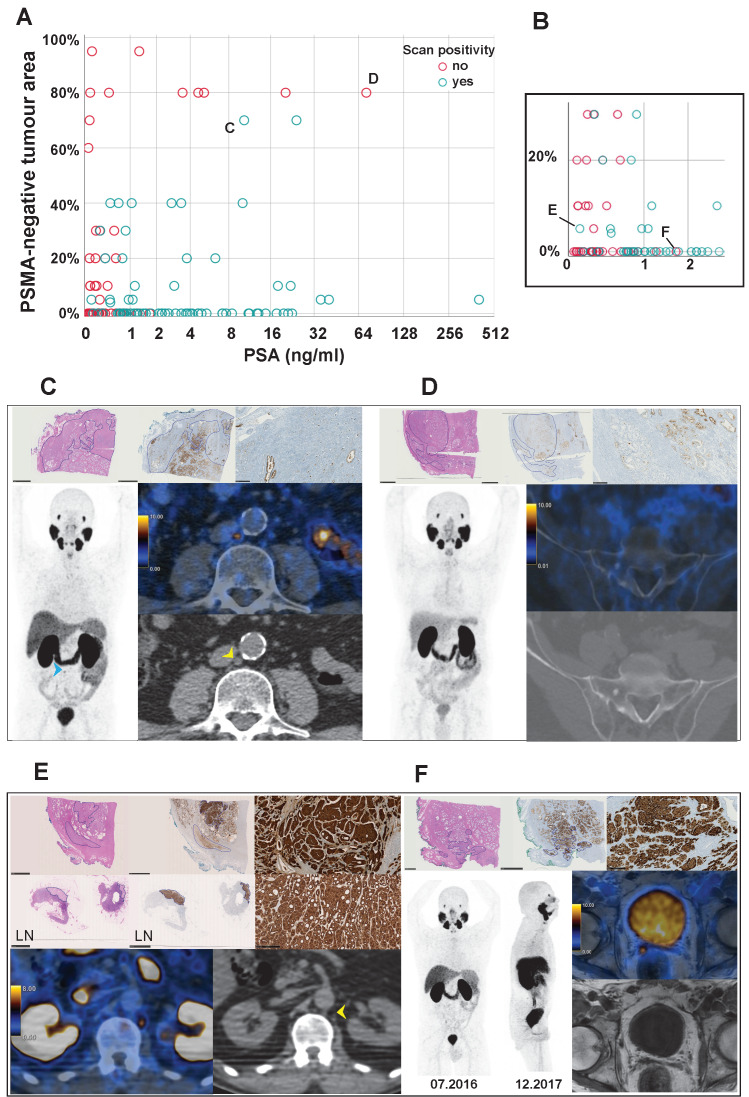
** (A)** Distribution of ^68^Ga-PSMA-11-PET scans according to primary tumour PSMA-negative tumour area (PSMA_%neg_) on immunohistochemistry (IHC) and patient prostate-specific antigen (PSA) level at scan time. PSMA_%neg_ is presented on linear scale and PSA level on logarithmic scale. Patients with a PSMA_%neg_ of 80% or more always had negative ^68^Ga-PSMA-11-PET scans, regardless of PSA levels. Patients with a PSMA-negative tumour area < 80% and a PSA level ≥ 2 ng/ml always had positive ^68^Ga-PSMA-11-PET scans. **(B)** Detail from figure A magnifying on scans from patients with low PSMA_%neg_ and low PSA values. **(C-F)** H&E (top left) and PSMA-IHC (top middle and magnification on top right) of the primary tumour and lymph nodes (only in figure E, middle row), as well as ^68^Ga-PSMA-11-PET images (maximum intensity projection (MIP), fused PET/CT or PET/MR and CT or MR) of the patients who had scans marked with the respective letters in figures A or B. MIP images are in the same intensity as the fused PET images. In the histopathology images, bars represent 5mm with tumour outlined in blue and 250 µm in the magnification of the PSMA-IHC: **(C)** Primary tumour (pT3b, GS 4+4 = 8) of a 57 y.o. patient with 70% PSMA_%neg_. ^68^Ga-PSMA-11-PET performed for biochemical recurrence at a PSA level of 10.4 ng/ml shows only two retroperitoneal lymph nodes with 7mm (arrowhead, SUV_max_ 7.4) and 5mm (not shown). The patient received radiotherapy (RT), with a partial drop of PSA and an immediate further rise, allowing the hypothesis that ^68^Ga-PSMA-11-PET underestimated disease. **(D)** Primary tumour (pT2c, GS 4+4 = 8) of a 68 y.o. patient with 80% PSMA_%neg_. The patient had two negative ^68^Ga-PSMA-11-PET scans for biochemical recurrence under PSA levels of 4.65 ng/ml and 20.36 ng/ml, respectively, and refused treatment. A third ^68^Ga-PSMA-11-PET scan was performed under a PSA level of 72 ng/ml and showed a new sclerotic lesion in the sacrum (arrowhead), suspicious for metastasis in the clinical context despite missing PSMA-expression, and confirmed by MR (supplementary Figure S3) which showed also multiple lesions in the vertebral spine and iliac bones. **(E)** Primary tumour (pT3a, GS 4+3 = 7) and a metastatic lymph node (LN) of a 64 y.o patient with 5% and 0% PSMA_%neg_, respectively. ^68^Ga-PSMA-11-PET performed for biochemical recurrence under a PSA level of 0.11 ng/ml with two small retroperitoneal nodes (SUV_max_ 7.8, pointed by the arrowhead, and 6.2, not shown). The patient underwent RT for the paraortal lymphatic chain achieving an undetectable PSA until last follow up. **(F)** Primary tumour (pT3b, GS 4+4 = 8) of a 64 y.o. patient with 0% PSMA_%neg._^68^Ga-PSMA-11-PET was negative despite a PSA of 1.69 ng/ml (only MIP shown). Patient underwent PSA follow-up and a second scan was performed when PSA level achieved 4.63 ng/ml, showing local recurrence in the seminal vesicles bed (blue arrowhead, SUV_max_ 7.3).

On a scan-based analysis, PSMA_%neg_ correlated with scan negativity (r = 0.309, *p* = 0.001, CI: 0.136, 0.482). The distribution of positive and negative ^68^Ga-PSMA-11-PET scans according to PSMA_%neg_ of the primary tumour and PSA level is shown in Figure 5 (A and B). A PSMA_%neg_ of 50% or more could predict a negative ^68^Ga-PSMA-11-PET with a positive predictive value (PPV) of 85% (11/13 scans were negative, in 9 patients). Only one patient with PSMA_%neg_ of 70% had positive scans (two), in both scans the PSMA-positive metastases were small, despite high PSA values of 10.4 ng/ml and 24 ng/ml (Figure 5C). By increasing the threshold to 80%, PPV would be 100% in our sample (9/9 scans were negative, in 6 patients, Figure 5D and supplementary Figure S3), without any patient presenting with a positive scan. Furthermore, all patients with a PSA level above 2 ng/ml and less than 80% PSMA_%neg_ had a positive ^68^Ga-PSMA-11-PET. For PSA values below 2 ng/ml in patients with a PSMA_%neg_ below 50%, scans were positive in 49% (33/68, Figure 5E) and negative in 51% (35/68). Nine of these 35 patients had a second scan, which was positive in five patients (median PSA: 1.32 ng/ml, IQR 1.2-3.37, Figure 5F) and again negative in four patients (median PSA: 0.55 ng/ml, IQR 0.4-0.76).

On a patient-based analysis including only one scan per patient (74 scans, chosen the one with the highest PSA value for patients with more than one scan), PSMA_%neg_ also correlated with scan negativity (r = 0.342, *p* = 0.003, CI: 0.121, 0.563). PPV for PSMA_%neg_ ≥ 50% and ≥ 80% was 88.8% and 100%, respectively.

## Discussion

Investigating clinical parameters and the dominant lesion of the primary tumour with PSMA-IHC, we found that ^68^Ga-PSMA-11-PET positivity in the BCR setting of PCa is associated with the PSA level at scan time, ongoing ADT and PSMA_%neg_ of the primary tumour.

Indeed, in patients with PSA values above 1 ng/ml and a dominant PSMA_%neg_ of 50% or more, ^68^Ga-PSMA-11-PET were either negative or likely underestimating the tumour burden as shown in Figure 5C, where two small lymph nodes were the only PSMA-positive findings at a PSA of 10.4 ng/ml. None of these tumours showed neuroendocrine differentiation.

PSMA-negativity is not an established IHC parameter for prostate cancer yet. However, the importance of prostate cancer heterogeneity and the high correlation between more than 50% positive tumour cells on PSMA-IHC with SUV_max_ of the primary tumour on ^68^Ga-PSMA-PET imaging has already been shown by Woythal et al 31. Our results suggest that this correlation between PSMA_%neg_ of the primary tumour with ^68^Ga-PSMA-PET imaging is maintained for metastasis in the BCR setting. In our cohort, the simple grading of intensity of membranous or cytosolic PSMA-expression was not associated with ^68^Ga-PSMA-11-PET positivity.

Several studies investigated potential clinical factors to predict a positive PSMA-PET for BCR. The best predictive factors reported so far are PSA level and ADT 16, which we could confirm as the only clinical parameters associated with PSMA-PET positivity in our cohort. However, even in patients with high PSA levels, the rate of positive PSMA-PET scans of around 90% probably implies that around 10% of PCa are PSMA-negative 16, without any clinical factor that can predict this.

Interestingly, molecular analysis of primary and recurrent PCA by Paschalis et al. also showed that the PSMA expression seems to be higher in lesions after ADT 9, which has also been confirmed with *in vitro* studies 32. Therefore, we believe that a combination of clinical parameters (PSA level and ADT) with quantitative PSMA-IHC of the primary tumour could be used to prevent repeated negative PSMA-PET scans.

Given that PCa can be a heterogeneous disease, and that PSMA-expression is associated with more aggressive PCa histology, it seems reasonable to not exclude patients from a first PSMA-PET scan for BCR. As in our cohort a patient with PSMA_%neg_ of 60% had a metastatic lymph node with PSMA_%neg_ of 0% (Pat.69 in Table 3), there is evidence by others that primary tumour and metastasis are not always concordant regarding PSMA-expression 1, 11, 33. Nevertheless, in patients who underwent RPE and had a first negative PSMA-PET performed for BCR, a high PSMA_%neg_ of the primary tumour is reducing the probability of PSMA-PET positivity. This limits the justification to further postpone blind salvage radiation in the hope to localize disease on later PSMA-PET scans, especially in patients with PSA levels of 1 ng/ml or more, as shown in Figure 5A-B. Of note is that the correlation between higher aggressiveness of tumour lesions and high PSMA expression is not correct in every stage of the disease. Results from the LuPSMA trial showed that in late stage castration-resistant prostate cancer patients with PSMA-negative, FDG-positive lesions did worse 34.

In this regard, PSMA-IHC analysis of the primary tumour might be an interesting tool to learn more about the specific disease of an individual patient and to evaluate the potential of PSMA-PET on a personal base. This might improve the individualized disease approach and avoid further negative scans.

The low number of primary tumours with significant PSMA-negative areas (9 patients with PSMA_%neg_ ≥ 50%) is the main limitation of our study. Therefore, it was not possible to give a definitive optimal threshold of percentage of PSMA_%neg_ for the likelihood of a negative ^68^Ga-PSMA-11-PET, which would have to be assessed in future studies with larger number of patients. The use of both PET/CT and PET/MR might have introduced some bias in the SUV_max_ measures as there is some variability among different PET scanners 35, though we believe it would not substantially affect the results or alter our conclusion. The use of ^68^Ga-PSMA-11 that is excreted over the kidneys might also be a potential limitation for PET/CT, evaluation of local recurrence. This was minimized with the use of diuretics. Furthermore, immediate applicability of our results is limited by the lack of assessment of PSMA-expression in clinical routine in prostatectomy specimens and, therefore, more research needs to be done to standardize the evaluation of PSMA_%neg_. However, the future potential of semiautomatic assessment of IHC with digital pathology will increase the possibility to incorporate these molecular parameters into decision making for patient selection.

## Conclusions

PSMA-negative tumour area of the primary tumour is associated with negative PSMA-PET scans for BCR. The integration of quantitative PSMA-expression based on the molecular pathology of the primary tumour with clinical parameters (PSA and ADT) has the potential to improve the patient selection and avoid further negative PET scans.

## Supplementary Material

Supplementary imaging protocols, contingency tables, statistical analysis, and supplementary figures.

## Figures and Tables

**Table 1:** Patient's characteristics (*n = 74*). Age and PSA are presented in years and ng/ml, respectively.

ISUP/WHO grade groups* (n = 74)	
1	1 (1%)
2	6 (8%)
3	14 (19%)
4	24 (33%)
5	29 (39%)
pT stadium (n = 74)	
pT2	22 (30%)
pT3	51 (69%)
pT4	1 (1%)
pN stadium (n = 74)	
pN0	44 (59%)
pN1	30 (41%)
At RPE (n = 74)	
Age median (IQR)	65 (60-70)
PSA median (IQR)	12 (7-19)
<10	33 (45%)
10-20	23 (31%)
>20	18 (24%)
At ^68^Ga-PSMA-11 PET scan time point (n = 120)**	
Age median (IQR)	69 (65-73)
ADT	17 (14%)
PSA median (IQR)	0.99 (0.32-4.27)
≤0.5	42 (35%)
0.5-10	59 (49%)
≥10	19 (16%)

*from radical prostatectomy (RPE) specimen **34 patients had two or three ^68^Ga-PSMA-11 PET scans

**Table 2:** Histopathological characteristics including androgen receptor (AR) and synaptophysin expression of the primary tumour of the five patients with PSA > 1 ng/ml and PSMA_%neg_ > 50%, six patients with homogeneous primary tumours without any PSMA_%neg_ and analysis for lymph nodes for AR, synapthophysin and Chromogranin A of the two patients shown in Figure 4.

Primary Tumour					
Patient	**PSMA_%neg_**	**Heterogeneity (+ or -)**	**AR**	**Synaptophysin**	**Morphology**
Pat.14	80%	**+**	2	single cells (<1%)	fused, poorly defined glands, no cribriform
Pat.19	80%	**+**	2	single cells (<5%)	fused, poorly defined glands, solid and single cells, cytoplasmic vacuoles, no cribriform
Pat.31	95%	**+**	1	0	single, acinary atypical glands, no cribriform
Pat.59	70%	**+**	2	single cells (<5%)	fused, poorly defined glands, prominent secretions, cribriform glands (<10%)
Pat.62	80%	**+**	3	single cells (<1%)	fused, cribriform glands (~50%), solid, single cells, partially intraductal
Pat.5	0%	**-**	2	single cells (<5%)	
Pat.10	0%	**-**	1	single cells (<5%)	
Pat.29	0%	**-**	0	0	
Pat.32	0%	**-**	1	single cells (<1%)	
Pat.42	0%	**-**	0	single cells (<5%)	
Pat.71	0%	**-**	1	single cells (<5%)	
					
Lymph Node					
Patient	**PSMA_%neg_**	**Heterogeneity (+ / -)**	**AR**	**Synaptophysin**	**Chromogranin A**
Pat.55	0%	**-**	1	single cells (<1%)	single cells (<1%)
Pat.45	90%	**+**	3	single cells (<1%)	0

AR** =** Androgenreceptor (quantified using a four-tiered system (0 = negative, 1+ = weak, 2+ = moderate, 3+ = strong); PSMA_%neg_ = PSMA-negative tumour area

**Table 3:** Patient-based comparison (n = 30) between PSMA-expression on IHC of primary tumours and lymph nodes. The right column shows an overview of positive (+) and negative (-) ^68^Ga-PSMA-11-PET scans the patient had according to PSA level at scan time (≤ 2 or > 2 ng/ml).

	PSMA-cytosol*		PSMA-membranous*		PSMA_%neg_		PSMA PET scan status (+ or -)	
	Primary tumour	Lymph node	Primary tumour	Lymph node	Primary tumour	Lymph node	PSA ≤ 2 ng/ml	PSA > 2 ng/ml
Pat.1	3	1	2	2	4%	0%	**+**	
Pat.2	3	2	3	3	0%	0%	**+**	
Pat.4	2	2	3	3	0%	0%	**-**	
Pat.5	2	1	3	3	0%	0%		**+ +**
Pat.7	2	2	3	3	0%	0%	**+**	**+ +**
Pat.12	2	2	2	2	20%	0%	**+**	**+**
Pat.16	3	2	3	3	0%	0%	**-**	
Pat.23	2	2	2	3	0%	0%	**- + +**	
Pat.27	2	1	2	2	10%	0%		**+**
Pat.32	2	2	3	3	0%	0%		**+**
Pat.33	3	2	3	2	5%	10%	**+**	
Pat.34	3	2	3	3	0%	0%	**-**	
Pat.36	1	1	3	3	0%	0%	**+**	
Pat.38	2	2	3	2	10%	10%	**-**	
Pat.39	1	2	3	3	5%	0%	**-**	
Pat.40	2	2	3	3	0%	0%	**- -**	
Pat.42	2	2	3	3	0%	0%		**+ +**
Pat.44	2	1	2	2	0%	0%	**- + +**	
Pat.45	2	2	2	3	80%	90%	**- - -**	
Pat.50	3	2	3	3	0%	0%	**-**	
Pat.51	3	2	3	3	0%	0%	**-**	
Pat.52	3	2	3	3	5%	0%	**+ +**	
Pat.54	3	2	3	3	40%	0%		**+ +**
Pat.55	2	2	3	2	0%	0%	**+**	
Pat.62	2	2	2	2	80%	70%		**-**
Pat.64	2	2	2	2	0%	0%		**+**
Pat.65	2	2	2	2	40%	40%	**+**	**+**
Pat.69	2	2	2	3	60%	0%	**-**	
Pat.70	3	2	2	3	0%	0%	**-**	
Pat.71	2	2	3	3	0%	0%		**+**

*quantified using a four-tiered system (0 = negative, 1+ = weak, 2+ = moderate, 3+ = strong) for PSMA-positive expression. IHC: immunohistochemistry. PSMA_%neg:_ percentage of PSMA-negative tumour area.

**Table 4:** Results of a multiple logistic regression model to predict negative PSMA PET scans

Variable	Estimate (log odds)	SE	p-value	OR	95% CI
(Intercept)	1.22	1.77	0.49	3.37	0.1 - 108.94
PSMA_memb_	-0.29	0.58	0.61	0.75	0.24 - 2.31
PSMA_cytosol_	-0.73	0.53	0.17	0.48	0.17 - 1.37
PSMA_%neg_ (multiples of 20%)	1.06	0.02	<0.001	2.88	2.8 - 2.97
ADT	-2.31	1.16	0.047	0.1	0.01 - 0.97
PSA_log_	-1.29	0.26	<0.001	0.28	0.17 - 0.46

ADT = androgen-deprivation therapy; CI = confidence interval; OR = odds ratio; PSA_log_ = PSA transformed to a natural logarithmic scale; PSMA_cytosol_ = PSMA-expression in the cytosol; PSMA_memb_ = PSMA-expression on the membrane; PSMA_%neg_ = PSMA-negative tumour area in the primary tumour; SE = standard error

## Abbreviations

ADT: antiandrogen therapy
AUC: area under the receiver operating curve
BCR: biochemical recurrence
CI: confidence interval
CT: computed tomography
GS: Gleason Score
IHC: immunohistochemistry
IQR: interquartile range
LN: lymph nodes
MIP: maximum intensity projection
MR: magnetic resonance
OR: odds ratio
PCa: prostate cancer
pLND: pelvic lymph node
PPV: positive predictive value
PSA: prostate-specific antigen
PSMA: prostate-specific membrane antigen
PSMA_cytosol_: cytoplasmic PSMA-expression
PSMA_memb_: membranous PSMA-expression
PSMA_%neg_: PSMA-negative tumour area on immunohistochemistry
PET: positron emission tomography
ROC: receiver operating curve
RPE: radical prostatectomy
RT: radiotherapy
SD: standard deviation
SUV_max_: maximum standard uptake value
