# Supplementary material for: Immunohistochemical PSMA expression patterns of primary prostate cancer tissue are associated with the detection rate of biochemical recurrence with 68Ga-PSMA-11-PET
Source: Theranostics. 2020 May 15;10(14):6082–94. doi: 10.7150/thno.44584 (PMC7255040; doi:10.7150/thno.44584)
Supplement: Supplementary file 1 — Supplementary imaging protocols, contingency tables, statistical analysis, and supplementary figures. [file thnov10p6082s1.pdf]

Supplementary Material

Imaging protocols

To reduce tracer activity in the bladder, ureters, and kidneys, furosemide was injected intravenously 30 min prior to the tracer injection (0.13 mg/kg), and patients were asked to void prior to the ^{68}Ga-PSMA-11 PET.

PET/MR protocol

A clinical routine whole-body PET/MR was performed 60 min after injection on a hybrid scanner (SIGNA PET/MR, GE Healthcare, Waukesha, WI, USA). PET acquisition for the whole-body protocol was in 3D time of flight (TOF) mode, six bed positions with 2-3 min acquisition time per bed position, an additional pelvic frame over 15 minutes was acquired but not used for quantification, axial FOV of 25cm and overlap of 24%, matrix 256x256, 2 iterations, 28 subsets, with sharpIR algorithm (GE Healthcare) and 5mm filter cutoff.

Details of MRI sequences are given in the table below:

	Axial DWI EPI (Focus) (Pelvis)	Axial LAVA-FLEX WB (DIXON)	Axial T1w Whole ARC (Pelvis)	Axial T2w FRFSE-XL (Pelvis)	Coronal T2w WB FRFSE-XL	Coronal T2w FRFSE-XL (Pelvis)	Axial DCE (Lava Dyn) (Pelvis)	Ax syn. DWI Focus	Sag T2w FRFSE
Repetition time, TR (ms)	4000	5.6	550	5034	5538	5034	6.361	3500	4678
Echo time, TE (ms)	67.3	1.3-2.7	8.26	120	120	120	2.376	Minimum	120
Flip angle, FA (degrees)	90	12	111	140	111	140	30	-	140
Acquisition matrix	160 x 80	344 x 256	384x384	300x280	288 x 224	300x280	160 x 80	140x70	300x272
Image size (voxels)	256 x 256	512 x 512	512 x 512	512 x 512	512 x 512	512 x 512	288 x 192	-	
Slice thickness (mm)	4	3	5	3.5	5	3.5	4	4	3.5
Signal averages	8	0.68	0.5	2	0.5	4	0.35	-	
b-values (s/mm2) and signal averages	0 (6 av.)<br>400 (8 av.)<br>700(16 av.)							0<br>400<br>1000<br>1500<br>2000	

Diffusion direction	‘All’							All	
Bandwidth (Hz/pixel)	1953	166	62.5	50	90.9	50	62.5	250	50
Acquisition time (mm:ss)	5:41	0:18	1:44	3:27	0:50	3:27	3:27	4:05	3:12

PET/CT protocol

Discovery VCT 690 PET/CT (GE Healthcare, Waukesha, WI, USA) and Discovery MI PET/CT (GE Healthcare, Waukesha, WI, USA)

PET was acquired in 3D TOF mode, with six bed positions with 2.5 min acquisition time per bed position, 23% overlap of bed positions and an axial FOV of 153 mm. The emission data were corrected for attenuation using CT and were iteratively reconstructed (matrix size of 256 x 256 pixels, 3D TOF ordered subset expectation maximization with 3 iterations and 16-18 subsets, with point spread function, 4.7 mm full width at half maximum, 1:4:1 weighted axial filtering). The attenuation CT scan was acquired with whole-body scan parameters as follows: tube voltage 140 kV, tube current with automated dose modulation with a maximum of 80 mA/slice, collimation 512 x 0.976, pitch 0.984:1, rotation time 0.5 s, coverage speed 78 mm/s, field of view (FOV) 50 cm, and images with a transverse pixel size of 0.976 and a slice thickness of 1.25 mm reconstructed in the axial plane.

Supplementary Material

Contingency Tables scan-based (n = 120)

TP = true positive

FP = false positive

TN = true negative

FN = false negative

Cut-off $PSMA_{\%neg}$ 50%

	Negative PSMA-PET scan	Positive PSMA-PET scan	Total
**≥50%** $PSMA_{\%neg}$	11 TP	2 FN	13
<**50%** $PSMA_{\%neg}$	35 FP	72 TN	107
Total	46	74	120 scans

Sensitivity: 24%

Specificity: 97%

PPV: 85%

NPV: 67%

Cut-off $PSMA_{\%neg}$ 80%

	Negative PSMA-PET scan	Positive PSMA-PET scan	Total
≥**80**% $PSMA_{\%neg}$	9 TP	0 FN	9
<**80**% $PSMA_{\%neg}$	37 FP	74 TN	111
Total	46	74	120 scans

Sensitivity: 20%

Specificity: 100%

PPV: 100%

NPV: 67%

Supplementary Material

Immunohistochemical PSMA expression patterns of primary prostate cancer tissue are associated with the detection rate of biochemical recurrence with ^{68}Ga-PSMA-11-PET.

Logistic Regression Analysis

Variables were included in the logistic regression analysis as follows:

- ISUP/WHO grade groups, obtained from the radical prostatectomy specimens, dichotomized in two ISUP/WHO grade groups (1-3, and 4-5);
- PSA level at ^{68}Ga-PSMA-11 PET scan time point, transformed to a natural logarithmic scale to correct the skewed distribution and because of a suspicion of non-linear effect;
- ADT, dichotomized as positive or negative. Patients who had ongoing ADT at scan time point were considered as positive;
- Primary tumour PSMA membranous staining on immunohistochemistry. PSMA-expression intensity (visual ordinal scale: 0, 1+, 2+ or 3+);
- Percentage of PSMA-negative tumour area (per multiples of 20%)
- Primary tumour PSMA cytoplasmic staining on IHC (visual ordinal scale: 0, 1+, 2+ or 3+);
- Injected ^{68}Ga-PSMA-11 dose, per multiples of 50 MBq;
- Patient age at scan time per multiples of 10 years.

Results of the Univariate Analysis

Variable	Estimate (log odds)	SE	p-value	OR	95% CI
**$PSMA_{memb}$**	-0.14	0.08	0.07	0.87	0.74 - 1.01
**$PSMA_{cytosol}$**	-0.16	0.08	0.04	0.85	0.74 - 0.99
**$PSMA_{\%neg}$ (multiples of 20%)**	0.12	0.008	<0.001	1.13	1.12 - 1.13
**Age (multiples of 10)**	-0.07	0.01	0.35	0.94	0.92 - 0.95
**Tracer injected dose (multiples of 50 MBq)**	0.02	0.003	0.87	1.02	1.01 - 1.02
**ADT**	-0.38	0.12	0.003	0.69	0.54 - 0.87
**PSA_{log}**	-0.15	0.02	<0.001	0.86	0.83 - 0.9
**ISUP/WHO grade group 4+5**	-0.08	0.11	0.46	0.92	0.75 - 1.14

ADT=androgen-deprivation therapy; CI=confidence interval; OR=odds ratio; $PSA_{log=}$PSA transformed to a natural logarithmic scale; $PSMA_{cytosol=}$PSMA-expression in the cytosol; $PSMA_{memb=}$PSMA-expression on the membrane; $PSMA_{\%neg}$=PSMA-negative tumour area in the primary tumour; SE= standard error

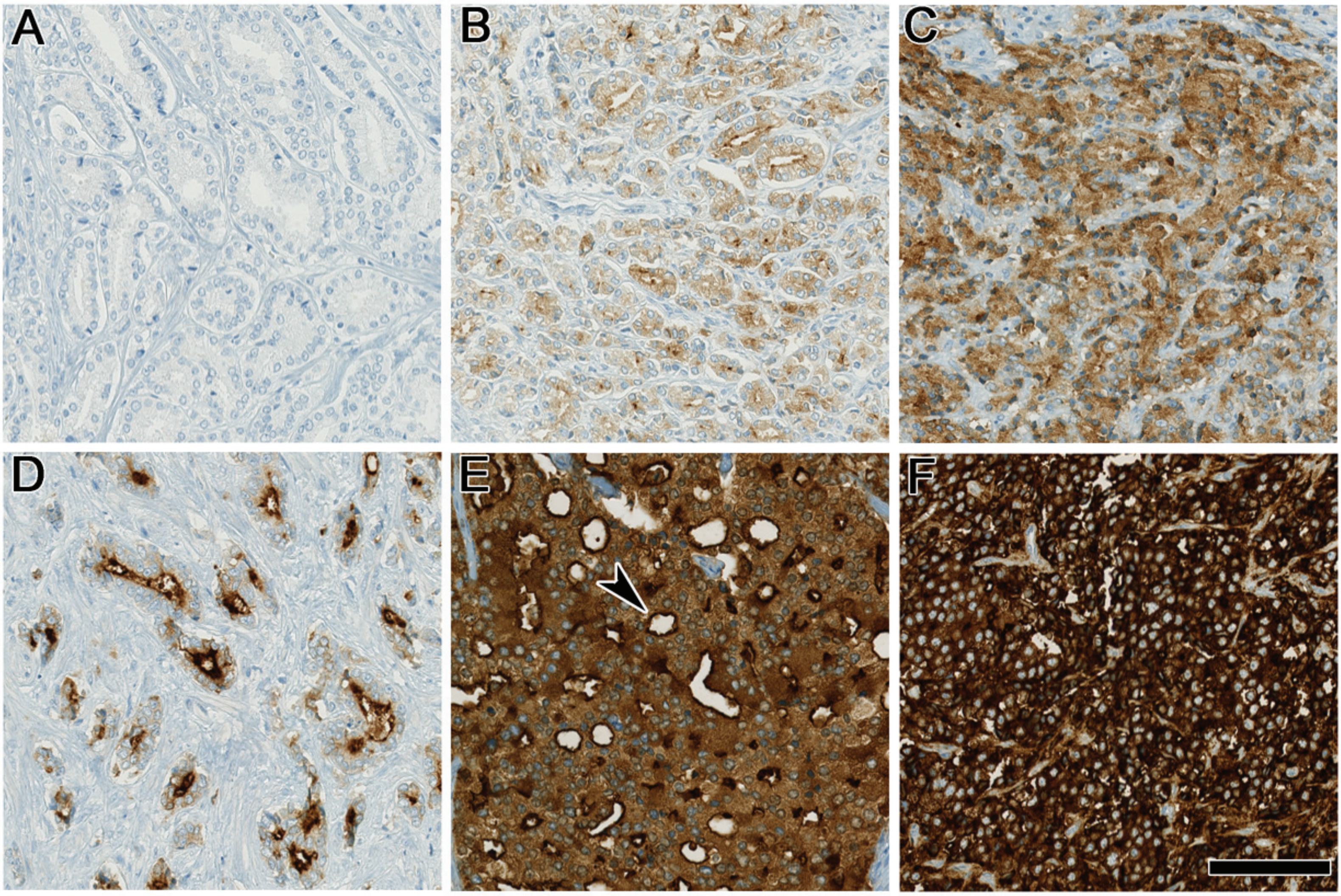

Figure S1. Overview of the different immunohistochemical PSMA staining patterns. (A) shows complete negativity, while (B) depicts low expression of cytoplasmic and membranous PSMA. In (C) a moderate cytoplasmic staining is shown. (D) illustrates low cytoplasmic and strong membranous (luminal) expression. (E) shows moderate cytoplasmic and strong membranous (luminal) expression (arrowhead), while (F) shows diffuse strong cytoplasmic and membranous expression. Scale bar 100 mu.

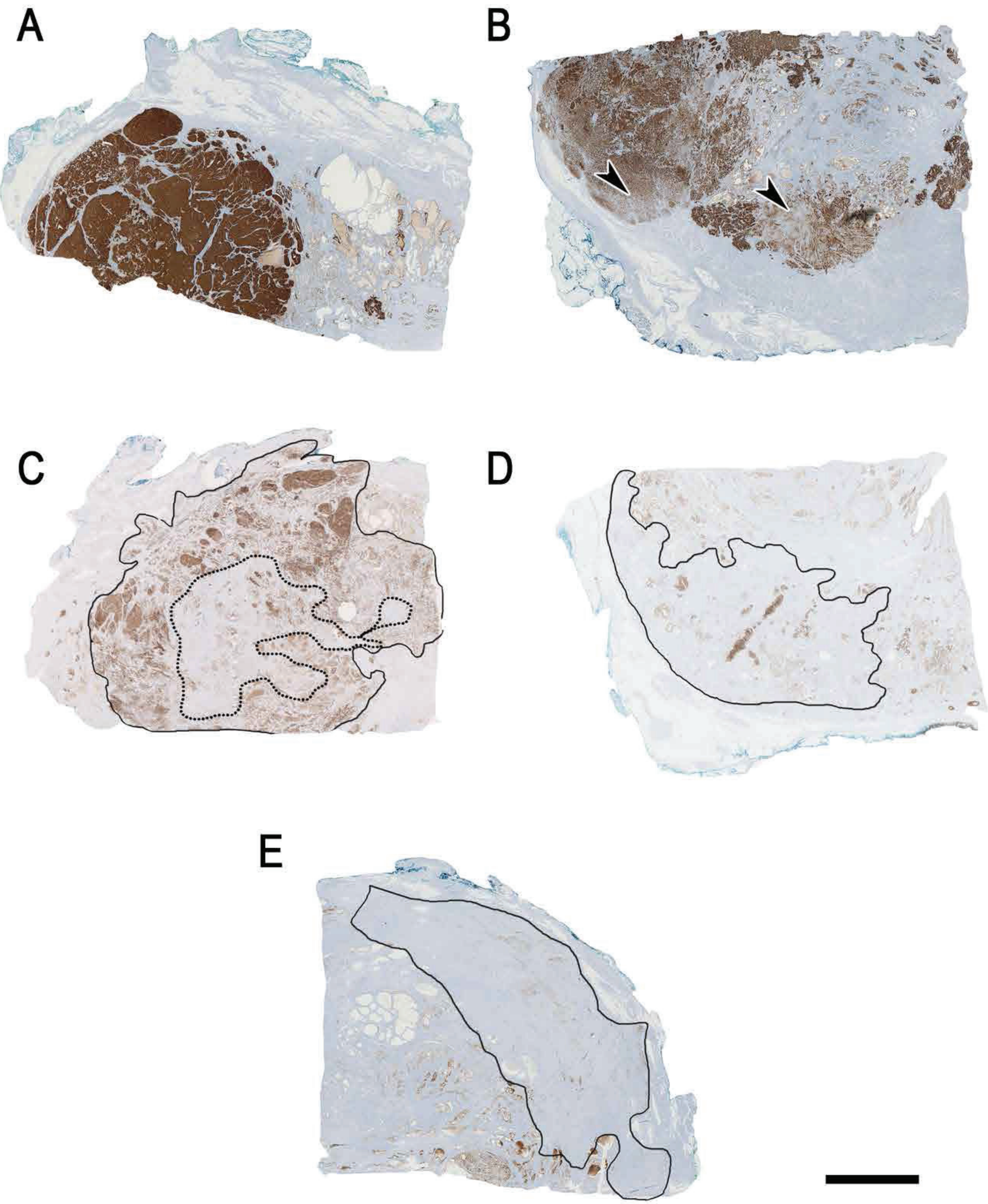

Figure S2. Overview of the different immunohistochemical PSMA heterogeneity patterns. (A) shows homogenous strong and diffuse positivity. (B) depicts heterogenous PSMA-positivity with focal weaker expression (arrowheads) in different components of the carcinoma, without negative areas. In (C) the circled carcinoma (continuous line) consists of approximately 40% (dotted line) negative areas. Whereas in (D) roughly 80% of the marked invasive carcinoma shows completely negative parts. In (E) the delineated carcinoma reacts almost completely negative for PSMA. Scale bar 5 mm.

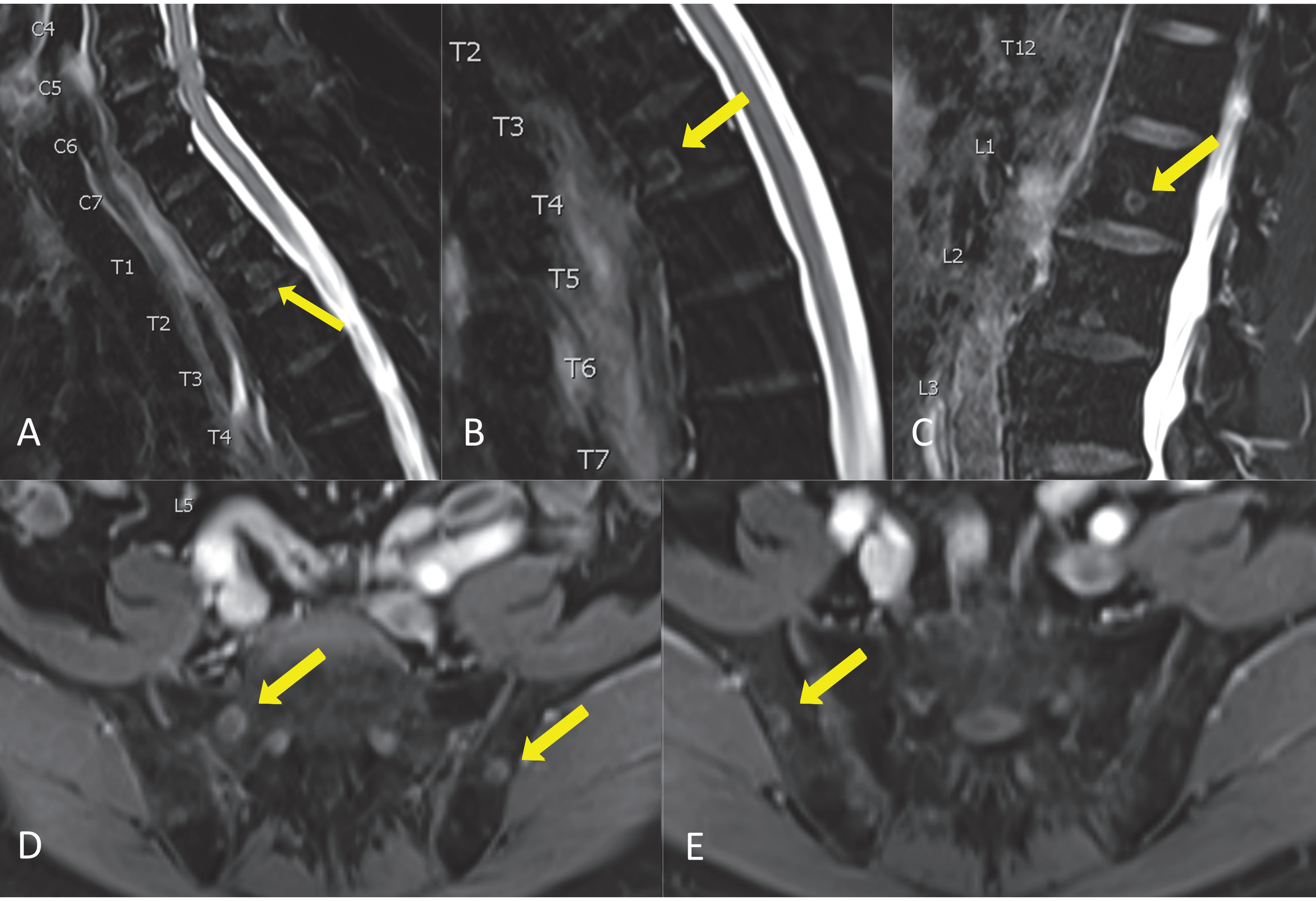

Figure S3 Whole-body magnetic resonance imaging (MRI) sagittal T2-weighted images with fat saturation shows multiple bone metastasis in the vertebral spine (arrows) in A, B and C and in the pelvic bone (arrows) in D and E (axial Dixon T1-weighted images post-contrast) in a a 68 y.o. patient with biochemical recurrence of prostate cancer (primary tumour pT2c, GS 4+4=8, 80% PSMA%neg). The patient had three negative 68Ga-PSMA-11-PET scans for biochemical recurrence under PSA levels of 4.65 ng/ml, 20.36 ng/ml and 72 ng/ml with a PSMA-negative suspicious new bone lesion in the sacrum in the latter scan.
